# Supplementary material for: Intrinsic brain connectivity after partial sleep deprivation in young and older adults: results from the Stockholm Sleepy Brain study
Source: Sci Rep. 2017 Aug 25;7:9422. doi: 10.1038/s41598-017-09744-7 (PMC5573389; doi:10.1038/s41598-017-09744-7)
Supplement: Supplementary file 1 — Supplementary material [file 41598_2017_9744_MOESM1_ESM.pdf]

Supplemental Information to:

Intrinsic brain connectivity after partial sleep deprivation in young and older adults: results from the Stockholm Sleepy Brain study

Gustav Nilsonne[1,2,*], Sandra Tamm[1,2], Johanna Schwarz[1,2], Rita Almeida[3], Håkan Fischer[4], Göran Kecklund[1], Mats Lekander[1,2], Peter Fransson[2], Torbjörn Åkerstedt[1,2]

1: Stockholm University, Stress Research Institute
2: Karolinska Institutet, Department of Clinical Neuroscience
3: Karolinska Institutet, Department of Neuroscience
4: Stockholm University, Department of Psychology

*Corresponding: gustav.nilsonne@ki.se

Supplemental table 1
Coordinates for seed regions based on de Havas et al. 2012.

		Talairach			MNI		
		x	y	z	x	y	z
DMN	PCC	1	-50	26	1	-29	49
	dmPFC	0	53	16	1	59	7
	vmPFC	0	50	-4	1	54	-14
	L IPL	-45	-66	31	-47	-66	37
	R IPL	45	-61	25	50	-61	29
	L LTC	-62	-7	-8	-66	-7	-12
	R LTC	57	-9	-14	62	-9	-20
ACN	L Insula	-39	5	7	-41	7	3
	R Insula	44	2	10	49	5	5
	L TPJ	-57	-31	34	-60	-28	37
	R TPJ	54	-31	34	60	-28	35
	L IPS	-27	-49	49	-27	-46	55
	R IPS	27	-43	43	31	-40	47

Supplemental table 2

Effects of KSS and sleep parameters on global signal variability in full sleep and sleep deprivation conditions. Because the sleep measures were so different between the full sleep and sleep deprived condition, regression models including both conditions are prone to unreliable results due to linear separation. Therefore, all covariates have been analysed separately in each condition, as well as in a full model including both conditions. Thus, the first column (Full sleep) reports the effect of each predictor in the full sleep condition only. The second column (Sleep deprived) reports the effect of each predictor in the sleep deprived condition only, and the last column (Interaction) reports the difference between conditions in the relation between each predictor and global signal variability. The table reports main effects as well as age group interaction effects, indicating estimated differences between older and younger participants in the relation of each predictor to the global signal. Thus, for example, the "KSS-age group interaction" row and the "Interaction" column give the estimated effect of increasing 1 step on the KSS scale and moving from the younger to the older group. Estimates are on the original scale, as illustrated in figure 7. For main effects of age group, see main text.

	**Full sleep**		**Sleep deprived**		**Interaction**	
	**Estimate [CI]**	***p***	**Estimate [CI]**	***p***	**Estimate [CI]**	***p***
**KSS main effect**	-0.008 [-0.046, 0.03]	0.68	0.005 [-0.04, 0.051]	0.82	0.01 [-0.018, 0.039]	0.48
**KSS-age group interaction**	0.006 [-0.071, 0.082]	0.88	-0.076 [-0.166, 0.014]	0.10	-0.043 [-0.089, 0.003]	0.06
**TST main effect**	0 [-0.001, 0.002]	0.55	-0.005 [-0.009, -0.001]	0.01	-0.002 [-0.004, -0.001]	0.00
**TST-age group interaction**	0 [-0.002, 0.003]	0.73	0.002 [-0.006, 0.01]	0.57	0.001 [0, 0.002]	0.00
**REM main effect**	0.003 [-0.012, 0.017]	0.70	-0.002 [-0.015, 0.011]	0.72	0 [-0.008, 0.008]	0.92
**REM-age group interaction**	0.01 [-0.019, 0.039]	0.49	0.003 [-0.024, 0.03]	0.81	0.01 [-0.004, 0.025]	0.16
**Stage 1 main effect**	-0.009 [-0.02, 0.002]	0.10	0.007 [-0.008, 0.022]	0.37	-0.006 [-0.015, 0.002]	0.13
**Stage 1-age group interaction**	0.012 [-0.01, 0.035]	0.27	0.009 [-0.021, 0.039]	0.54	0.015 [-0.001, 0.03]	0.06
**Stage 2 main effect**	-0.001 [-0.014, 0.013]	0.94	0.001 [-0.008, 0.009]	0.87	-0.001 [-0.007, 0.005]	0.81
**Stage 2-age group interaction**	-0.014 [-0.041, 0.014]	0.32	-0.004 [-0.02, 0.013]	0.67	-0.003 [-0.014, 0.008]	0.61
**Stage 3 main effect**	0 [-0.012, 0.012]	0.98	-0.003 [-0.01, 0.005]	0.51	0.001 [-0.005, 0.007]	0.79
**Stage 3-age group interaction**	0 [-0.024, 0.025]	0.99	-0.001 [-0.017, 0.015]	0.87	-0.003 [-0.012, 0.005]	0.48
**Stage changes main effect**	-0.014 [-0.026, -0.002]	0.02	0.008 [-0.006, 0.022]	0.24	-0.006 [-0.015, 0.002]	0.12
**Stage changes interaction**	-0.004 [-0.029, 0.02]	0.73	-0.011 [-0.038, 0.016]	0.40	-0.005 [-0.021, 0.012]	0.58

**Delta power (absolute) main effect**	0.026 [-0.261, 0.313]	0.86	0.068 [-0.134, 0.269]	0.50	0.023 [-0.132, 0.179]	0.77
**Delta power (absolute) interaction**	-0.004 [-0.575, 0.568]	0.99	0.271 [-0.131, 0.674]	0.18	0.137 [-0.178, 0.452]	0.39
**Delta power (relative) main effect**	-0.368 [-2.079, 1.344]	0.67	-0.841 [-2.182, 0.501]	0.21	-0.68 [-1.653, 0.293]	0.17
**Delta power (relative) interaction**	1.061 [-2.443, 4.566]	0.54	0.478 [-2.186, 3.141]	0.72	0.861 [-1.067, 2.788]	0.38
**Number of awakenings main effect**	-0.011 [-0.022, 0.001]	0.06	0.001 [-0.029, 0.032]	0.92	-0.002 [-0.015, 0.01]	0.71
**Number of awakenings interaction**	0.01 [-0.012, 0.033]	0.36	0.03 [-0.03, 0.09]	0.32	0.016 [0.004, 0.029]	0.01
**Sleep efficiency main effect**	0.01 [-0.006, 0.026]	0.20	0.01 [-0.008, 0.029]	0.26	0.005 [-0.006, 0.015]	0.36
**Sleep efficiency interaction**	-0.022 [-0.054, 0.01]	0.18	-0.035 [-0.072, 0.001]	0.06	-0.024 [-0.045, -0.003]	0.03
**Wake after sleep onset main effect**	-0.003 [-0.007, 0]	0.08	-0.006 [-0.017, 0.005]	0.26	-0.004 [-0.008, 0]	0.08
**Wake after sleep onset interaction**	0.008 [0.001, 0.016]	0.03	0.016 [-0.005, 0.037]	0.13	0.008 [0.003, 0.013]	0.00

Supplemental table 3

Effects of KSS and sleep parameters on global signal in full sleep and sleep deprivation conditions. Estimates are on the original scale, as illustrated in supplemental figure 6.

	**Full sleep**		**Sleep deprived**		**Interaction**	
	**Estimate [CI]**	***p***	**Estimate [CI]**	***p***	**Estimate [CI]**	***p***
**KSS main effect**	11.019 [-5.16, 27.198]	0.18	6.93 [-7.69, 21.55]	0.35	2.705 [-11.959, 17.368]	0.72
**KSS-age group interaction**	24.871 [-7.371, 57.114]	0.13	-4.06 [-32.935, 24.816]	0.78	20.034 [-2.767, 42.836]	0.08
**TST main effect**	0.214 [-1.23, 1.657]	0.77	-0.461 [-4.176, 3.254]	0.80	-0.673 [-1.351, 0.006]	0.05
**TST-age group interaction**	0.919 [-1.962, 3.801]	0.52	4.162 [-3.265, 11.589]	0.26	0.117 [-0.235, 0.469]	0.51
**REM main effect**	-4.71 [-19.447, 10.027]	0.52	-5.386 [-16.908, 6.136]	0.35	-1.998 [-6.441, 2.445]	0.38
**REM-age group interaction**	1.813 [-27.712, 31.338]	0.90	5.053 [-18.064, 28.169]	0.66	-4.499 [-12.109, 3.11]	0.24
**Stage 1 main effect**	-3.08 [-13.602, 7.443]	0.56	-4.418 [-17.009, 8.172]	0.48	0.522 [-4.006, 5.05]	0.82
**Stage 1-age group interaction**	-6.154 [-27.216, 14.908]	0.56	6.008 [-19.17, 31.186]	0.63	-1.916 [-10.411, 6.579]	0.66
**Stage 2 main effect**	-3.376 [-16.065, 9.313]	0.59	-2.208 [-9.061, 4.644]	0.52	1.721 [-1.411, 4.853]	0.28
**Stage 2-age group interaction**	-8.618 [-33.991, 16.756]	0.50	-4.287 [-17.992, 9.419]	0.53	0.097 [-5.043, 5.236]	0.97
**Stage 3 main effect**	162.638 [-135.257, 460.533]	0.28	4.262 [-2.014, 10.538]	0.18	2.443 [-0.688, 5.574]	0.12
**Stage 3-age group interaction**	35.056 [-560.269, 630.381]	0.91	1.598 [-11.007, 14.203]	0.80	1.29 [-2.601, 5.181]	0.51
**Stage changes main effect**	-5.187 [-16.834, 6.46]	0.37	-3.565 [-14.723, 7.592]	0.52	-1.905 [-6.303, 2.493]	0.39
**Stage changes interaction**	-9.507 [-32.889, 13.876]	0.42	12.909 [-9.399, 35.218]	0.25	0.522 [-8.306, 9.349]	0.91
**Delta power (absolute) main effect**	162.638 [-135.257, 460.533]	0.28	101.581 [-83.574, 286.736]	0.27	19.854 [-77.077, 116.786]	0.69
**Delta power (absolute) interaction**	35.056 [-560.269, 630.381]	0.91	304.738 [-65.555, 675.031]	0.10	-9.161 [-206.362, 188.04]	0.93
**Delta power (relative) main effect**	590.949 [-765.7, 1947.599]	0.38	208.296 [-323.694, 740.285]	0.43	246.414 [-89.07, 581.897]	0.15
**Delta power (relative) interaction**	-0.047 [-0.176, 0.082]	0.46	-0.012 [-0.056, 0.031]	0.57	119.46 [-576.5, 815.421]	0.73

**Number of awakenings main effect**	-6.472 [-17.035, 4.092]	0.22	-4.581 [-29.27, 20.107]	0.71	1.217 [-5.056, 7.491]	0.70
**Number of awakenings interaction**	-6.252 [-27.328, 14.825]	0.55	14.064 [-35.274, 63.403]	0.57	-4.11 [-9.75, 1.53]	0.15
**Sleep efficiency main effect**	2.688 [-12.318, 17.694]	0.72	14.347 [-0.683, 29.378]	0.06	-5.934 [-11.253, -0.615]	0.03
**Sleep efficiency interaction**	8.439 [-21.558, 38.435]	0.57	-30.541 [-60.611, -0.47]	0.05	15.943 [5.317, 26.57]	0.00
**Wake after sleep onset main effect**	-1.057 [-4.708, 2.593]	0.56	-8.044 [-16.487, 0.398]	0.06	1.045 [-1.049, 3.139]	0.32
**Wake after sleep onset interaction**	-0.165 [-7.457, 7.128]	0.96	14.15 [-2.713, 31.012]	0.10	-3.639 [-5.791, -1.488]	0.00
**ISI main effect**	-7.842 [-57.492, 41.808]	0.75	-12.273 [-54.849, 30.303]	0.57	-6.691 [-49.998, 36.617]	0.76
**ISI interaction**	-9.004 [-108.194, 90.186]	0.86	-24.389 [-109.511, 60.734]	0.57	-11.677 [-98.227, 74.872]	0.79
**ESS main effect**	-7.841 [-29.895, 14.213]	0.48	-11.935 [-30.565, 6.695]	0.20	-9.076 [-28.169, 10.016]	0.34
**ESS interaction**	24.678 [-19.433, 68.789]	0.27	30.72 [-6.53, 67.97]	0.10	26.266 [-11.913, 64.445]	0.17
**KSQ sleep quality main effect**	16.846 [-151.744, 185.436]	0.84	22.569 [-121.143, 166.28]	0.75	9.971 [-136.473, 156.414]	0.89
**KSQ sleep quality interaction**	-116.908 [-453.692, 219.876]	0.49	-168.563 [-455.595, 118.469]	0.24	-145.014 [-437.483, 147.454]	0.32

Supplemental figure 1: Masks for resting state networks of interest

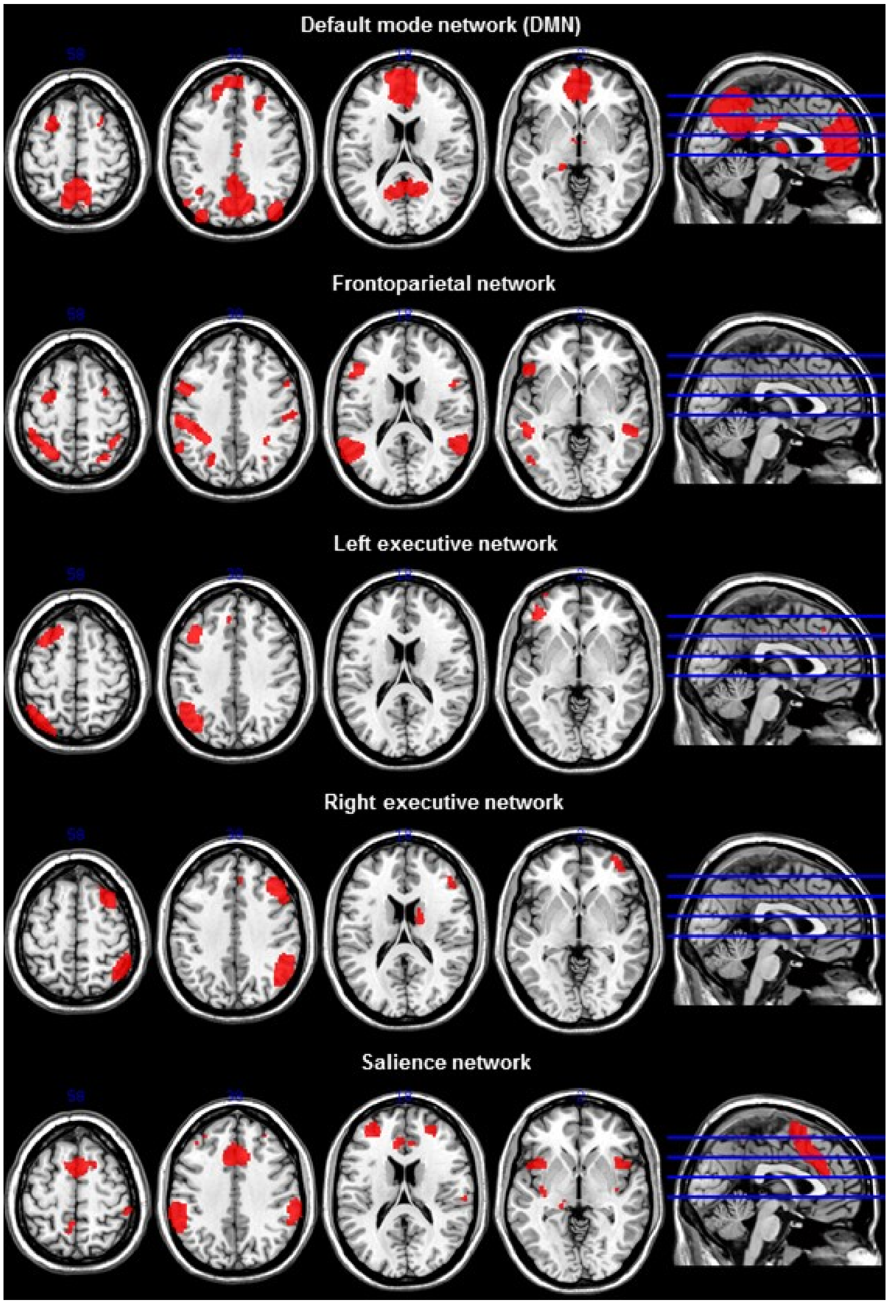

Networks were defined based on a parcellation published previously by Shirer et al. (2012).

Supplemental figure 3: Thalamus seed region

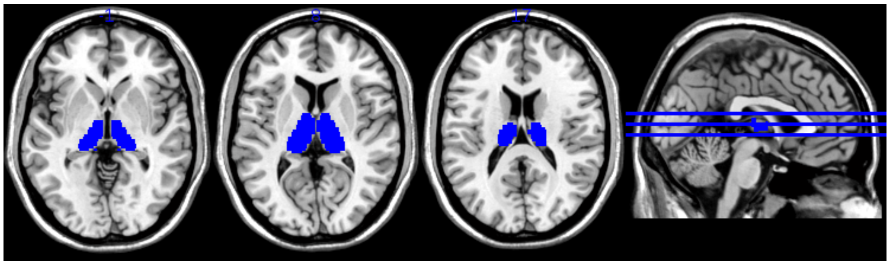

Seed region for bilateral thalamus, defined from the Wake Forest University PickAtlas, following Shao et al., 2013.

Supplemental figure 4: Amygdala seed regions

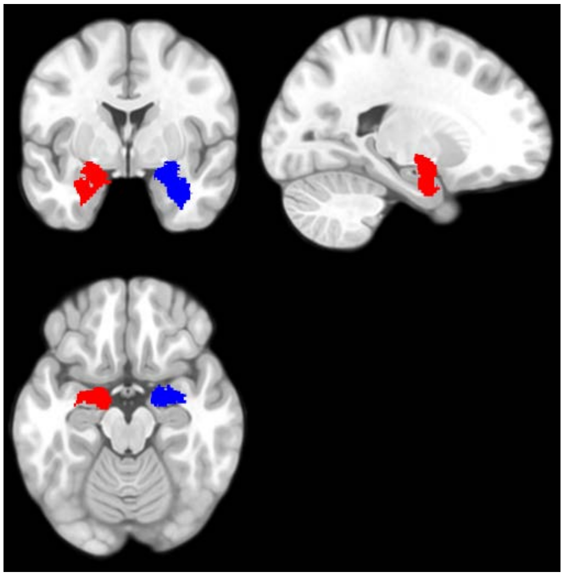

Seed regions for left and right amygdala, defined from the Jülich Atlas.

Supplemental figure 5: Thalamus connectivity

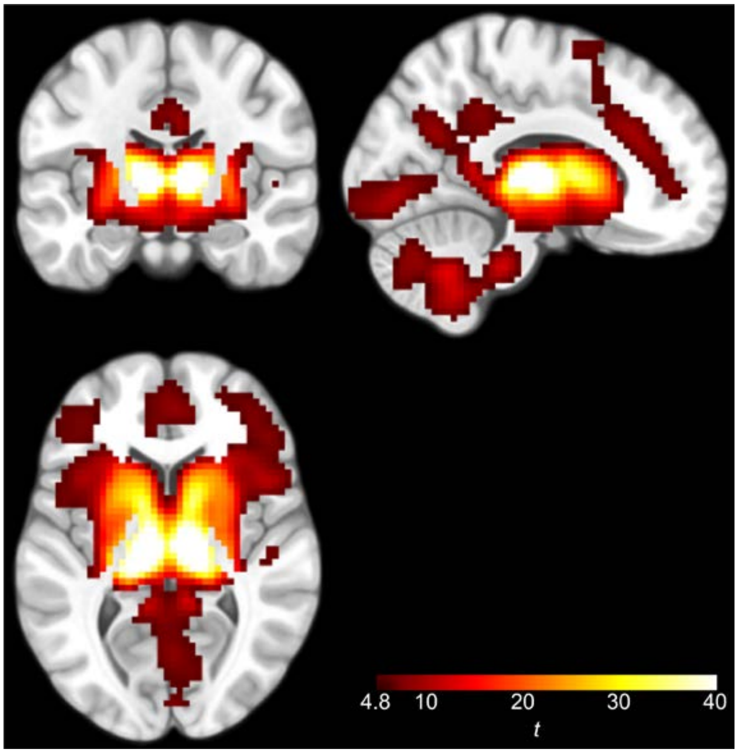

Thalamus connectivity across conditions and groups. Scale truncated at *t* = 40.

Supplemental figure 6: Amygdala connectivity

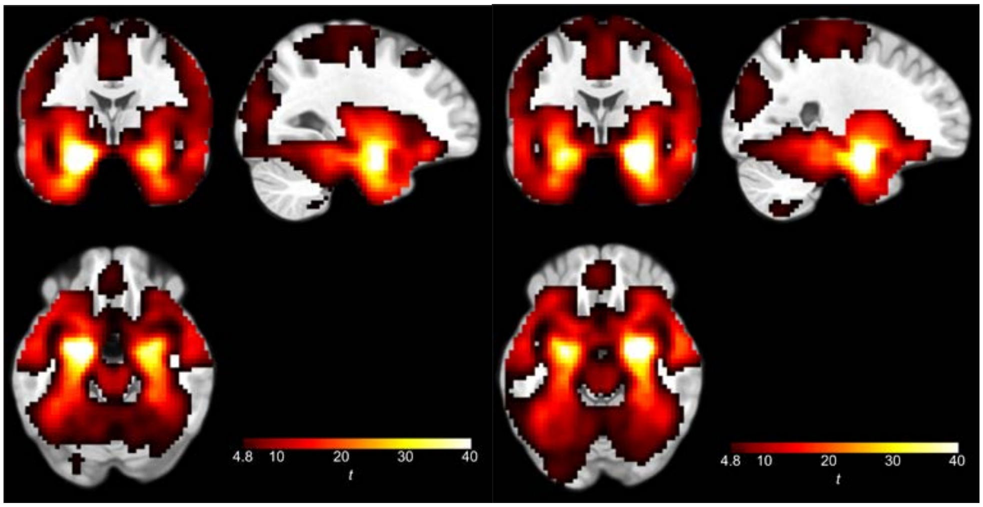

Left and right amygdala connectivity across conditions and groups. Scale truncated at *t* = 40.

Supplemental figure 7: Default mode network seed regions

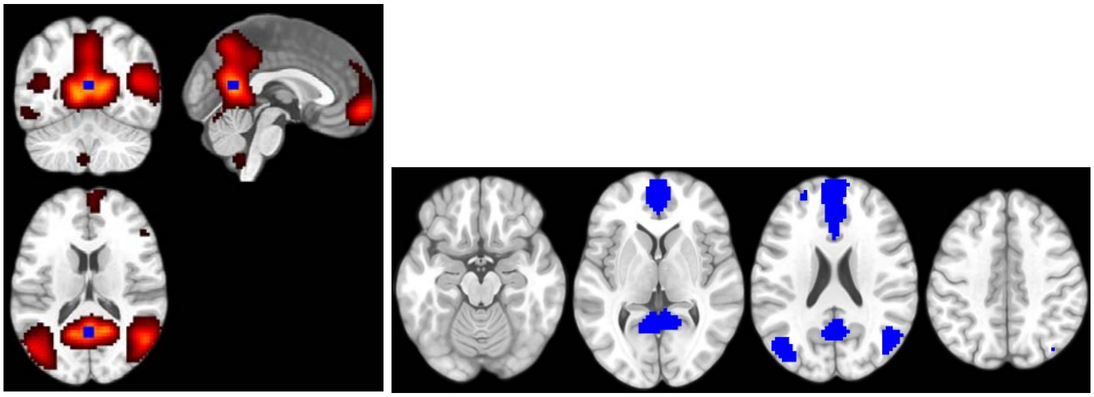

Left: Box seed centered on top coordinate of posterior default mode network (component 18, see figure 2 of main manuscript); component 18 is visualized in the same image. Right: Seed regions based on posterior and anterior components of the DMN (components 18 and 19, see figure 2 of main manuscript).

Supplemental figure 8: Default mode network connectivity

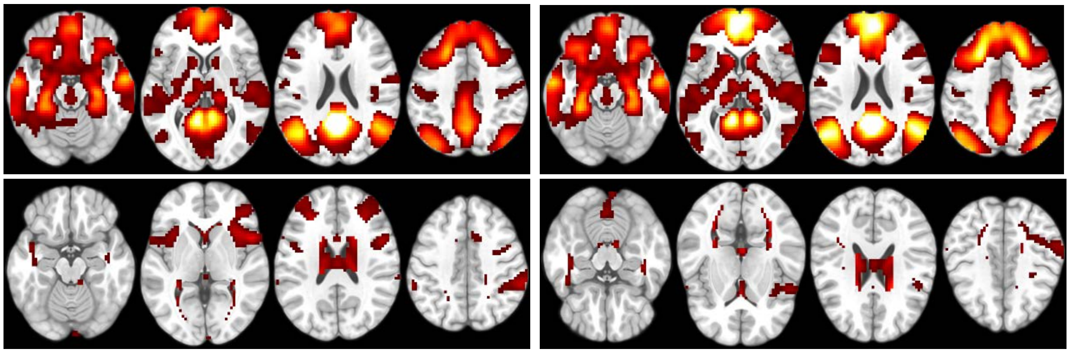

Top left: Positive connectivity from box seed. Top right: Positive connectivity from multiple seed regions. Bottom left: Negative connectivity from box seed. Bottom right: Negative connectivity from multiple seed regions.

Supplemental figure 9: Global signal

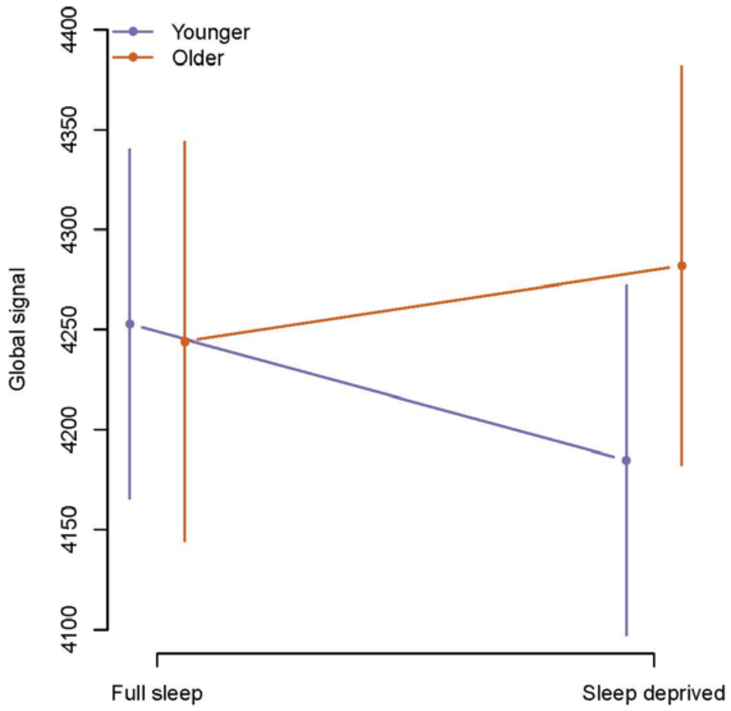

Global signal after sleep deprivation in younger and older participants. Error bars show 95% confidence intervals.
